# Development and external validation of an explainable machine learning model for identifying depressive symptoms in middle-aged and older adults with low haemoglobin: a prediction-model study

**DOI:** 10.3389/fnut.2026.1846276

**Published:** 2026-10-09

**Authors:** Qilong Wang, Juan He, Siheng Ma, Jianpeng Zhao, Jingjing Zong, Dongmei Ma, Sha Liu, Lei Zhang, Xin Qi, Dongrong Zhao

**Affiliations:** 1 Department of Psychiatry, Gansu Provincial People’s Hospital, The First Clinical College of Gansu University of Chinese Medicine, Lanzhou, Gansu, China; 2 Department of Psychiatry, Xijing Hospital, The Fourth Military Medical University, Xi’an, Shaanxi, China; 3 Department of Psychiatry, Baoji Rehabilitation Hospital, Baoji, Shaanxi, China

**Keywords:** depressive symptoms, low haemoglobin, machine learning, middle-aged and older adults, random forest, risk stratification

## Abstract

**Background:**

Early screening for depressive symptoms in middle-aged and older adults is important for timely risk identification. Low haemoglobin may identify a clinically accessible vulnerable subgroup in which chronic disease burden, fatigue, functional impairment, and reduced physiological reserve often coexist. However, it should be understood as a high-risk phenotype rather than a direct measure of nutritional deficiency or frailty. Evidence in this population remains limited.

**Methods:**

This study developed a model using data from the 2015 China Health and Retirement Longitudinal Study (CHARLS), with CHARLS 2011 and a cross-sectional cohort from Gansu Provincial People’s Hospital used for temporal and external validation, respectively. Depressive symptoms were defined as a 10-item Centre for Epidemiologic Studies Depression Scale score ≥10. Candidate variables included sociodemographic, behavioural, health status, functional, cognitive, and comorbidity-related information. After least absolute shrinkage and selection operator feature selection, 16 variables were retained. Seven machine learning models were compared, and Random Forest was retained for interpretation and online tool development based on its balanced performance and interpretability.

**Results:**

A total of 2,022 participants were included in the development cohort, 1,095 in the temporal validation cohort, and 400 in the external validation cohort. The AUROC values of the Random Forest model were 0.791, 0.776, and 0.809, respectively, with Brier scores of 0.182, 0.193, and 0.182, indicating stable discrimination and acceptable calibration. Shapley additive explanations analysis showed that sleep time, instrumental activities of daily living score, and activities of daily living score were the most important contributors. Threshold sensitivity analysis showed that lower probability thresholds increased sensitivity/recall but reduced specificity. A prototype online assessment tool was developed for individualised estimation of depressive symptom probability.

**Conclusion:**

This study developed and validated an interpretable machine learning model for identifying depressive symptoms in middle-aged and older adults with low haemoglobin. The model may support early screening and risk stratification in this clinically vulnerable population. However, the classification threshold and risk-stratification cut-offs are exploratory rather than clinically validated. Further multicentre validation is warranted before broader clinical implementation.

## Graphical abstract

**Figure:**
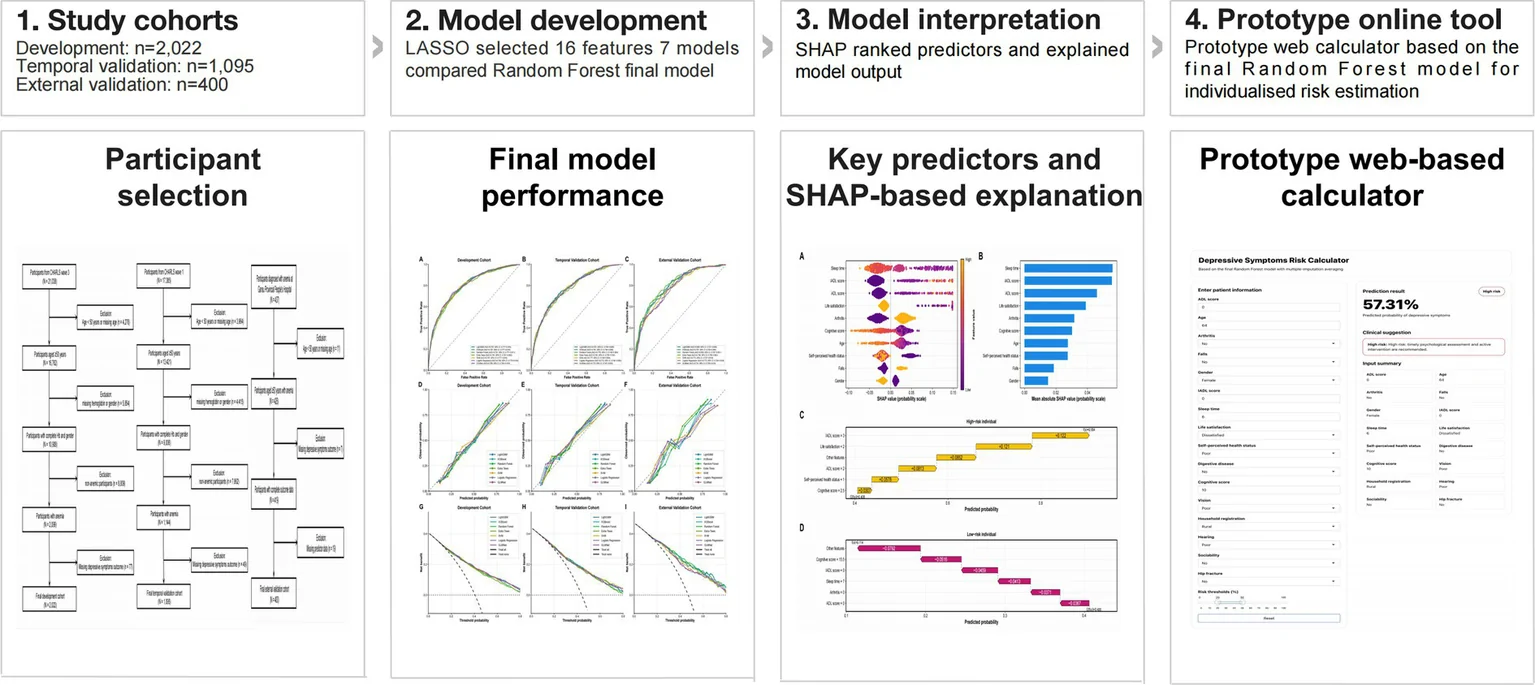

## 1 Introduction

Depressive symptoms are one of the most common mental health issues among middle-aged and older adults, and are closely associated with reduced quality of life, functional impairment, difficulties in managing chronic conditions, and adverse health outcomes (1–3). Among middle-aged and older adults, low haemoglobin is commonly observed and is clinically relevant from both nutritional and geriatric perspectives, as it may be associated with nutritional deficiency, chronic disease burden, inflammation-related processes, and potential nutritional vulnerability (4–6). Although haemoglobin level is not a direct measure of nutritional status, inflammation, or frailty, it is a routinely measured and clinically accessible indicator that can help define a potentially vulnerable subgroup. Previous studies have shown a significant association between anaemia or low haemoglobin and depressive symptoms, and this association is of particular concern in community-dwelling older adults (7, 8). However, depression in older adults is often under-recognised in routine clinical and community settings, and barriers to screening may delay timely identification and intervention (9, 10). From a nutrition-related risk identification perspective, depressive symptoms in middle-aged and older adults with low haemoglobin may coexist with multiple demographic, nutritional vulnerability-related, functional, cognitive, comorbidity-related, and psychosocial factors (4–6, 11, 12). Recent evidence further supports the link between metabolic dysfunction, chronic disease burden, and depression: higher cardiovascular-kidney-metabolic syndrome stages have been associated with increased depression risk (13), metabolic syndrome has been linked to a growing burden of depressive symptoms in middle-aged and older adults (14), and adverse metabolic biomarker profiles have been associated with future risks of depression, anxiety, and stress-related disorders (15). Together, these findings suggest that middle-aged and older adults with low haemoglobin may represent a clinically and nutritionally relevant vulnerable population in whom depressive symptom identification may benefit from a multivariable risk-prediction approach.

Although machine learning has increasingly been applied to depression identification, several gaps remain. First, previous machine learning studies have examined depression identification in disease-specific populations, such as individuals with hypertension or diabetes (16, 17). In contrast, to our knowledge, the subgroup of middle-aged and older adults with low haemoglobin has received less attention from a nutrition-related risk-identification and stratification perspective. Second, traditional statistical methods, such as multivariable logistic regression, remain widely used in medical prediction research (18). Although these methods offer good interpretability and clinical applicability, misspecification of functional forms, especially for continuous predictors, may affect model performance, and traditional modelling strategies may be less flexible in capturing complex non-linear patterns and interactions (19). By contrast, machine learning methods offer advantages in modelling high-dimensional data, capturing complex relationships, and identifying variable interactions in epidemiological research (20). In addition, interpretable machine learning approaches may improve model transparency and facilitate clinical understanding of prediction results (21). Furthermore, previous machine learning studies in depression have raised concerns regarding predictive validity and generalisability, highlighting the need for validation across independent cohorts or external clinical settings (22, 23).

Based on this, the present study used the China Health and Retirement Longitudinal Study (CHARLS) database and a hospital-based cohort from Gansu Provincial People’s Hospital to develop and validate an interpretable machine learning model to identify depressive symptoms in middle-aged and older adults with low haemoglobin levels. Low haemoglobin was used to define a clinically accessible and nutrition-relevant target population for model development and validation, rather than as an exposure variable for causal or association analysis. Therefore, this study was not designed to test whether low haemoglobin causes depressive symptoms or to quantify the independent association between haemoglobin level and depressive symptoms. Instead, it focused on individuals with low haemoglobin as a clinically accessible high-risk subgroup and aimed to evaluate whether routinely available demographic, behavioural, functional, cognitive, and comorbidity-related information could be integrated to support the identification of depressive symptoms in this subgroup. We compared seven machine learning algorithms, applied Shapley additive explanations (SHAP) to interpret the final model, and developed a prototype online assessment tool to support individualised estimation of depressive symptom probability.

## 2 Methods

This study was reported in accordance with the Transparent Reporting of a Multivariable Prediction Model for Individual Prognosis or Diagnosis + Artificial Intelligence (TRIPOD-AI) guidelines for the development, internal validation, and external validation of prediction models (23).

### 2.1 Study participants

The development dataset used in this study was derived from the 2015 wave of CHARLS. CHARLS is a nationally representative longitudinal study of Chinese adults aged 45 years and older and collects extensive demographic, health, and socioeconomic information (24). Based on predefined inclusion and exclusion criteria, this study included participants aged 50 years or older and defined low haemoglobin according to the World Health Organization (WHO) criteria for anaemia in adults: haemoglobin <13.0 g/dL for men and <12.0 g/dL for women, with haemoglobin concentration expressed in g/dL. Using these sex-specific thresholds, we identified middle-aged and older adults with low haemoglobin from the 2015 CHARLS dataset to construct the development cohort. Low haemoglobin was used to define the target population for model development and validation, rather than as an exposure variable for causal or association analysis. To assess the model’s generalisability and robustness, we further included two independent datasets for validation: a temporal validation cohort derived from the 2011 CHARLS data and an external validation cohort recruited from Gansu Provincial People’s Hospital between February 2025 and February 2026.

In total, 2,022 participants were included in the development cohort, 1,095 in the temporal validation cohort, and 400 in the external validation cohort. Because this study was based on secondary analysis of CHARLS and an external hospital-based cohort, no formal *a priori* sample size calculation was performed; instead, all eligible participants who met the predefined inclusion and exclusion criteria were included. The adequacy of the sample size was considered with reference to current guidance for prediction model development and reporting, with attention to the number of participants, outcome events, and non-events (22). The development cohort included 822 participants with depressive symptoms and 1,200 without depressive symptoms. The temporal validation cohort included 481 participants with depressive symptoms and 614 without depressive symptoms, and the external validation cohort included 188 participants with depressive symptoms and 212 without depressive symptoms. The participant selection process is shown in Figure 1, and the sample size and outcome event distribution across cohorts are shown in Supplementary Table S1. The CHARLS study was approved by the Ethics Review Committee of Peking University, and all participants provided written informed consent. The hospital-based cross-sectional cohort study was approved by the Ethics Committee of Gansu Provincial People’s Hospital (Approval No. 2025-472), and all participants provided written informed consent before enrolment. Patients or members of the public were not involved in the design, conduct, reporting, or interpretation of this study. For the CHARLS cohort, this study was based on secondary analysis of publicly available, de-identified data. For the external hospital-based validation cohort, participants provided written informed consent and contributed clinical and questionnaire data, but they were not involved in the development of the research question, selection of outcomes, model development, interpretation of findings, or preparation of the manuscript.

**Figure 1:**
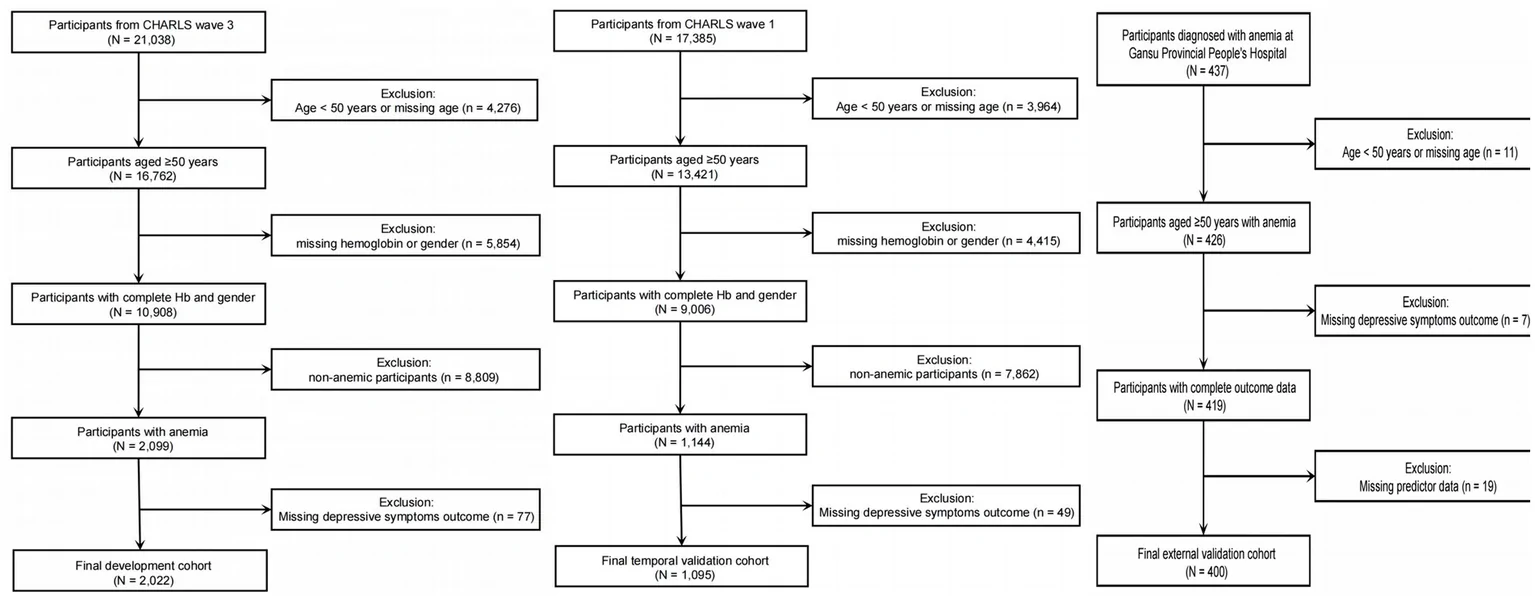
Flow diagram of participant selection.

### 2.2 Outcome definition

This study used the 10-item Centre for Epidemiologic Studies Depression Scale (CESD-10) to assess depressive symptoms (25). The CESD-10 comprises 10 items, each scored on a 4-point scale according to symptom frequency. The total score ranges from 0 to 30, with higher scores indicating more severe depressive symptoms. In line with the cut-off widely adopted in previous studies, a CESD-10 total score of ≥10 was defined as indicating the presence of depressive symptoms (26). The development cohort, temporal validation cohort, and external validation cohort were all assessed using the same CESD-10 instrument and the same cut-off value to identify depressive symptoms. Based on the CESD-10 scores, participants were classified into a depressive symptoms group and a non-depressive symptoms group.

### 2.3 Candidate predictors

The candidate predictors included in this study mainly comprised sociodemographic characteristics, behavioural characteristics, and health-related indicators. Sociodemographic characteristics included Age, Gender, Marital status, Household registration, Education, living with children, currently working, and Number of family members. Behavioural characteristics included Smoking status, Alcohol consumption, Internet use, Sociability, and Sleep time (hours). Health-related indicators included Hypertension, Dyslipidemia, Heart disease, Stroke, Liver diseases, Kidney disease, Digestive disease, Diabetes, Diabetes medication use, Cancer, Arthritis, Chronic lung disease, Falls, and Hip fracture. In addition, Life satisfaction, Vision, Hearing, Self-perceived health status, activities of daily living (ADL) score, instrumental activities of daily living (IADL) score, and Cognitive score were included. ADL was assessed using six items: dressing, bathing, eating, getting in and out of bed, using the toilet, and bowel and bladder control. IADL was assessed using five items: housework, cooking, shopping, making telephone calls, and taking medication. Each item was coded as present or absent, and the item scores were summed, with higher scores indicating more severe functional impairment (27). Cognitive function was assessed using the Cognitive score, which ranged from 0 to 21 and was derived from three components: Telephone Interview for Cognitive Status-10 (TICS-10), word recall, and figure drawing; higher scores indicated better cognitive function (28). More direct nutritional and biological indicators, including body mass index (BMI), serum albumin, ferritin, iron metabolism markers, inflammatory biomarkers, detailed dietary intake, and formal frailty scores, were not included because these variables were not simultaneously available across all three cohorts. Therefore, this study focused on routinely available sociodemographic, behavioural, functional, cognitive, and comorbidity-related predictors to enhance cross-cohort applicability and practical feasibility in primary care, with ADL, IADL, and falls serving as clinical proxies for frailty. Therefore, this study focused on routinely available sociodemographic, behavioural, functional, cognitive, and comorbidity-related predictors to enhance cross-cohort applicability and practical feasibility in primary care, with ADL, IADL, and falls serving as clinical proxies for frailty. Detailed definitions and coding of the candidate predictors are provided in Supplementary Table S2.

### 2.4 Data preprocessing and feature selection

The CHARLS 2015 cohort of middle-aged and older adults with low haemoglobin was defined as the development cohort, while the CHARLS 2011 cohort and the cross-sectional cohort from Gansu Provincial People’s Hospital served as the temporal validation cohort and the external validation cohort, respectively. Model development, feature selection, hyperparameter optimisation, and evaluation of model performance were all conducted within the development cohort, whereas the temporal validation cohort and external validation cohort were used solely to assess the model’s generalisability. As the external validation data were derived from a single-centre hospital-based cohort and did not involve a multi-centre or multi-cluster data structure, this study did not formally assess heterogeneity in model parameter estimates or model performance across centres or clusters. Model performance in the development cohort was evaluated using stratified 5-fold cross-validation, without further splitting into a separate internal test set. The decision not to split a separate internal test set was made to maximize the utilization of the development cohort data for model training, feature selection, hyperparameter optimisation, and internal performance assessment, while maintaining the outcome distribution within each fold. The generalisability of the final model was rigorously assessed through comprehensive temporal and external validation using the CHARLS 2011 temporal validation cohort and the hospital-based external validation cohort. Missing data were handled according to predefined rules. First, candidate variables with a missing rate greater than 20% were excluded from subsequent analyses. For the remaining missing values in the retained variables, multiple imputation by chained equations (MICE) was applied. To prevent information leakage, the imputation process was performed only within the training subsets of each fold in the stratified 5-fold cross-validation of the development cohort and was then applied to the corresponding validation subsets, as well as to the temporal validation cohort and the external validation cohort. Five imputed datasets were generated for each training subset (m = 5), with 10 iterations for each imputation (maxit = 10). Continuous variables were imputed using predictive mean matching, binary variables using logistic regression, and multiclass variables using multinomial logistic regression. Because there was no severe imbalance in the distribution of outcome categories in the development cohort, no class imbalance handling methods, such as the Synthetic Minority Over-sampling Technique (SMOTE), were applied during model development. Feature selection was performed using least absolute shrinkage and selection operator (LASSO) regression. LASSO applies a penalty to regression coefficients, shrinking some coefficients to zero and thereby selecting variables most relevant to the outcome. The penalty parameter λ was determined using 10-fold cross-validation, and the corresponding cross-validation curves and coefficient paths are shown in Figure 2. To enhance model interpretability, reduce feature dimensionality, and ensure comparability across different algorithms, LASSO regression was first used to select a unified set of candidate features, after which different machine learning models were constructed and compared using the same feature set. A total of 16 features were ultimately retained for subsequent modelling, including ADL, Age, Arthritis, Falls, Gender, IADL, Sleep time (hours), Life satisfaction, Self-perceived health status, Digestive disease, Cognitive score, Vision, Household registration, Hearing, Sociability, and Hip fracture. Considering the number of outcome events in the development cohort, the sample size was considered adequate relative to the final set of predictors used for model development. The Pearson correlation matrix of the final predictors in the development cohort is shown in Supplementary Figure S1. The variance inflation factors for the predictors included in the final Random Forest model are presented in Supplementary Table S3.

**Figure 2:**
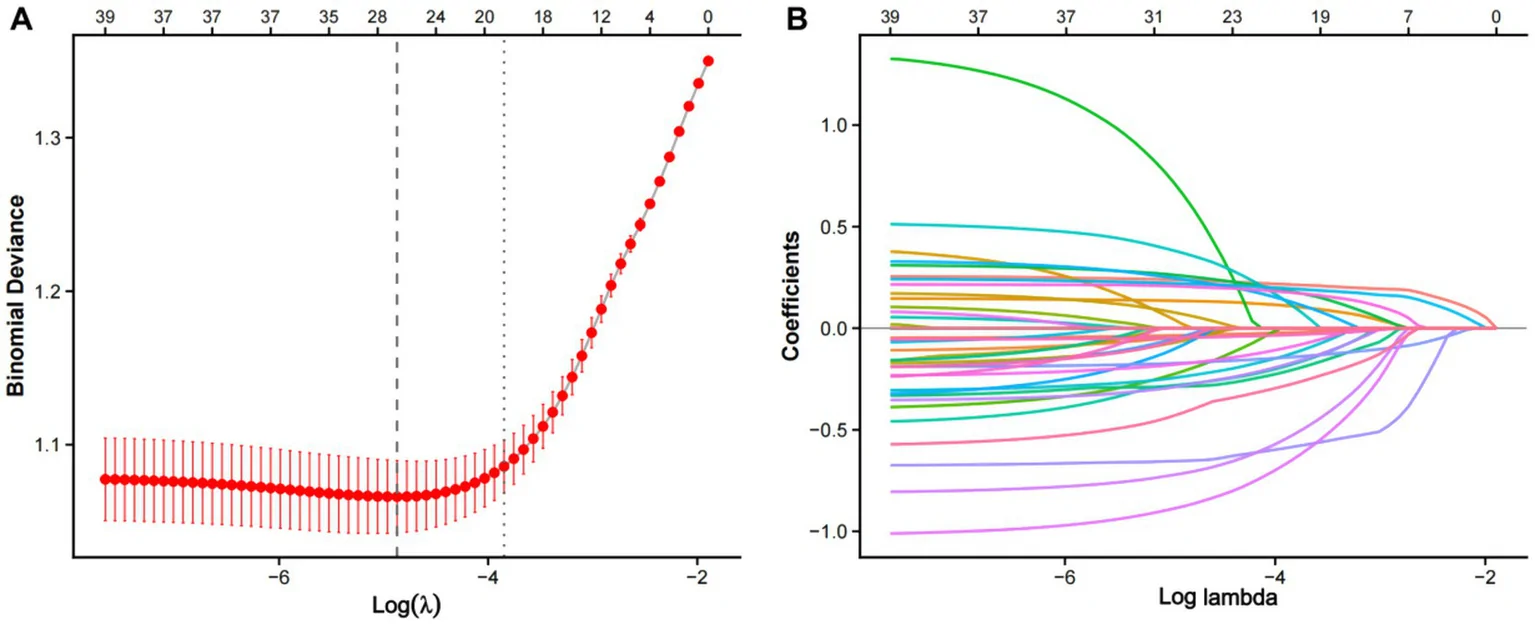
Feature selection using LASSO regression. **(A)** Ten-fold cross-validation determined the optimal penalty parameter (λ). The y-axis shows binomial deviance; the upper x-axis shows the number of non-zero coefficients. **(B)** LASSO coefficient profiles of candidate predictors against log (λ). Each colored line represents the coefficient trajectory of a predictor as λ changes.

### 2.5 Model development and evaluation

Based on the 16 selected features, this study constructed seven machine learning models to identify depressive symptoms in middle-aged and older adults with low haemoglobin, including Logistic Regression, support vector machine (SVM), XGBoost, LightGBM, Random Forest, Extra Trees, and GLMNet. For models requiring hyperparameter tuning, stratified 5-fold cross-validation was used for optimisation, with the area under the receiver operating characteristic curve (AUROC) as the primary tuning metric. All models were trained and tuned within the development cohort and were then validated separately in the temporal validation cohort and the external validation cohort. After model development was completed, these validation cohorts were used only to evaluate the locked candidate models and were not used for feature selection, hyperparameter tuning, model updating, or recalibration. The model output was the predicted probability of depressive symptoms for each participant. For the temporal validation cohort and the external validation cohort, predicted probabilities were generated for each imputed dataset, and the final predicted probability for each participant was calculated by averaging the predicted probabilities across imputations. For the calculation of accuracy, precision, recall, specificity, and F1 score, the default classification threshold of 0.5 was used to convert predicted probabilities into binary classification results. The 0.5 threshold was used as a conventional statistical threshold for reporting binary classification metrics and was not intended to represent a clinically validated decision threshold for depression screening. To evaluate threshold-dependent performance, a threshold sensitivity analysis was conducted for the final Random Forest model using probability thresholds of 0.20, 0.30, 0.40, and 0.50. At each threshold, accuracy, sensitivity/recall, specificity, precision/positive predictive value (PPV), negative predictive value (NPV), and F1 score were calculated to evaluate the trade-off between missed cases and false-positive classifications. Model performance was evaluated in terms of discrimination, calibration, and clinical utility. For final model reporting and prototype online tool development, discrimination, calibration, clinical utility, cross-cohort consistency, and model interpretability were considered together, rather than AUROC or Brier score in any single cohort alone. Discrimination was assessed using receiver operating characteristic (ROC) curves, AUROC, accuracy, precision, recall, specificity, F1 score, and the area under the precision–recall curve (AUPRC); calibration was assessed using calibration curves, the Brier score, calibration intercept, and calibration slope; and clinical utility was assessed using decision curve analysis (DCA) to evaluate net benefit across different threshold probabilities.

In addition, risk stratification analysis was conducted in the external validation cohort, and participants were categorised into low-risk (<0.20), medium-risk (0.20 to <0.50), and high-risk (≥0.50) groups according to predicted probability. Because no established risk-probability cut-off exists for this specific low-haemoglobin population, these categories were used for exploratory risk stratification and should not be interpreted as clinically validated diagnostic categories. The Pearson correlation matrix of the final predictors is shown in Supplementary Figure S1, the precision -recall curves are shown in Supplementary Figure S2, the confusion matrices are shown in Supplementary Figure S3, the distribution of predicted probabilities is shown in Supplementary Figure S4, the risk stratification results are shown in Supplementary Figure S5, and the threshold-dependent performance metrics are presented in Supplementary Table S4.

### 2.6 SHAP Explainability analysis

This study used the SHAP method to interpret the final Random Forest model. Based on a game-theoretic framework, SHAP quantifies the contribution of each predictor to the model output by calculating SHA*p* values. Using the full sample from the external validation cohort, we generated SHAP summary plots, feature importance plots, individual waterfall plots, and dependence plots to interpret the final model and to illustrate the direction and magnitude of each predictor’s contribution to the model output. SHA*p* values were used to improve model transparency and should not be interpreted as evidence of causal effects.

### 2.7 Development of a prototype online assessment tool

This study developed a prototype online assessment tool based on the final Random Forest model to estimate the probability of depressive symptoms in middle-aged and older adults with low haemoglobin. The tool incorporates the 16 predictors included in the final model. After the relevant clinical information is entered, the system automatically generates an individualised predicted probability of depressive symptoms together with the corresponding exploratory risk stratification result. The tool was designed to demonstrate the potential translation of the model into a pre-screening and risk stratification aid, rather than as a replacement for CESD-10 assessment or formal clinical diagnosis. Before real-world clinical implementation, the tool requires prospective validation, routine calibration monitoring, privacy and data-security assessment, and workflow evaluation to determine how clinicians or community health workers interpret and use the risk output in actual screening settings.

### 2.8 Statistical analysis

Participants were classified into a non-depressive symptoms group and a depressive symptoms group according to depressive symptom status. Continuous variables were expressed as mean ± standard deviation, and categorical variables as frequencies and percentages. Continuous variables were compared using one-way analysis of variance (ANOVA), and categorical variables using the chi-squared test; Fisher’s exact test was applied when expected cell counts were small. All data processing, model development, and statistical analyses were conducted in R software (version 4.5.2). A two-sided *p* value < 0.05 was considered statistically significant.

## 3 Results

### 3.1 Patient characteristics

The baseline characteristics of the Development Cohort, Temporal Validation Cohort, and External Validation Cohort are shown in Table 1. The mean ages of the three cohorts were 65.07, 64.83, and 63.48 years, respectively. Overall, the age distributions were similar, although the External Validation Cohort was relatively younger. Women accounted for a high proportion in all three cohorts, with the Development Cohort having the highest proportion of women (60.48%). Marked differences were observed across the cohorts in education level, Internet use, Sleep time, ADL score, IADL score, and several chronic diseases. Compared with the CHARLS development and temporal validation cohorts, the External Validation Cohort had a relatively higher educational level, higher Internet use, shorter Sleep time, higher ADL and IADL scores, and higher proportions of Hypertension, Dyslipidemia, Diabetes, Diabetes medication use, Arthritis, and Chronic lung disease. In contrast, the Temporal Validation Cohort had the lowest proportion of participants with Falls. No significant differences were observed in the cognitive score across the three cohorts. Overall, these differences indicate that the hospital-based external validation cohort represented a clinically distinct population rather than a simple replication of the CHARLS cohorts.

**Table 1:** Baseline characteristics of participants in the development, temporal validation, and external validation cohorts.

Variables	Development cohort (*n* = 2022)	Temporal validation cohort (*n* = 1,095)	External validation cohort (*n* = 400)	*p* value
Age (years), Mean ± SD	65.07 ± 9.30	64.83 ± 9.21	63.48 ± 6.77	0.006
Sleep time (hours), Mean ± SD	6.18 ± 2.12	6.14 ± 1.99	5.26 ± 2.04	<0.001
Number of family members, Mean ± SD	2.96 ± 1.37	3.53 ± 1.97	2.89 ± 1.18	<0.001
IADL score, Mean ± SD	0.58 ± 1.11	0.59 ± 1.17	0.76 ± 1.28	0.013
ADL score, Mean ± SD	0.53 ± 1.12	0.47 ± 1.10	0.68 ± 1.24	0.005
Cognitive score, Mean ± SD	9.31 ± 4.89	9.37 ± 4.35	9.12 ± 4.76	0.656
Gender, *n* (%)				0.003
Female	1,223 (60.48%)	597 (54.52%)	223 (55.75%)	
Male	799 (39.52%)	498 (45.48%)	177 (44.25%)	
Education, *n* (%)				<0.001
Below high school	1902 (94.07%)	1,036 (94.61%)	318 (79.50%)	
High school	108 (5.34%)	50 (4.57%)	55 (13.75%)	
College or above	12 (0.59%)	9 (0.82%)	27 (6.75%)	
Currently working, *n* (%)				0.14
No	735 (36.77%)	442 (40.37%)	150 (37.50%)	
Yes	1,264 (63.23%)	653 (59.63%)	250 (62.50%)	
Internet use, *n* (%)				<0.001
No	1975 (97.68%)	1,084 (99.00%)	350 (87.50%)	
Yes	47 (2.32%)	11 (1.00%)	50 (12.50%)	
Household registration, *n* (%)				0.004
Rural	1,341 (66.32%)	753 (68.77%)	238 (59.50%)	
Urban	681 (33.68%)	342 (31.23%)	162 (40.50%)	
Marital status, *n* (%)				0.409
Other marital status	358 (17.71%)	176 (16.07%)	74 (18.50%)	
Married or partnered	1,664 (82.29%)	919 (83.93%)	326 (81.50%)	
Living with children, *n* (%)				0.357
No	953 (47.65%)	549 (50.14%)	200 (50.00%)	
Yes	1,047 (52.35%)	546 (49.86%)	200 (50.00%)	
Smoking status, *n* (%)				<0.001
No	1,578 (78.12%)	789 (72.05%)	300 (75.00%)	
Yes	442 (21.88%)	306 (27.95%)	100 (25.00%)	
Alcohol consumption, *n* (%)				0.051
No	1,444 (71.45%)	757 (69.13%)	302 (75.50%)	
Yes	577 (28.55%)	338 (30.87%)	98 (24.50%)	
Life satisfaction, *n* (%)				<0.001
Not satisfied	39 (1.95%)	23 (2.10%)	30 (7.50%)	
Average	152 (7.58%)	124 (11.32%)	50 (12.50%)	
Satisfied	1814 (90.47%)	948 (86.58%)	320 (80.00%)	
Stroke, *n* (%)				0.91
No	1836 (96.43%)	1,056 (96.44%)	384 (96.00%)	
Yes	68 (3.57%)	39 (3.56%)	16 (4.00%)	
Sociability, *n* (%)				0.002
No	1,112 (55.00%)	666 (60.82%)	246 (61.50%)	
Yes	910 (45.00%)	429 (39.18%)	154 (38.50%)	
Liver diseases, *n* (%)				<0.001
No	1767 (93.64%)	1,060 (96.80%)	378 (94.50%)	
Yes	120 (6.36%)	35 (3.20%)	22 (5.50%)	
Kidney disease, *n* (%)				<0.001
No	1,676 (88.40%)	1,024 (93.52%)	346 (86.50%)	
Yes	220 (11.60%)	71 (6.48%)	54 (13.50%)	
Digestive disease, *n* (%)				<0.001
No	1,241 (65.45%)	853 (77.90%)	276 (69.00%)	
Yes	655 (34.55%)	242 (22.10%)	124 (31.00%)	
Hypertension, *n* (%)				<0.001
No	1,301 (68.80%)	835 (76.26%)	247 (61.75%)	
Yes	590 (31.20%)	260 (23.74%)	153 (38.25%)	
Dyslipidemia, *n* (%)				<0.001
No	1,528 (83.45%)	1,021 (93.24%)	309 (77.25%)	
Yes	303 (16.55%)	74 (6.76%)	91 (22.75%)	
Heart disease, *n* (%)				<0.001
No	1,571 (83.17%)	972 (88.77%)	339 (84.75%)	
Yes	318 (16.83%)	123 (11.23%)	61 (15.25%)	
Diabetes, *n* (%)				<0.001
No	1,675 (89.19%)	1,025 (93.61%)	338 (84.50%)	
Yes	203 (10.81%)	70 (6.39%)	62 (15.50%)	
Diabetes medication use, *n* (%)				<0.001
No	1713 (92.90%)	1,053 (96.16%)	349 (87.25%)	
Yes	131 (7.10%)	42 (3.84%)	51 (12.75%)	
Cancer, *n* (%)				0.157
No	1856 (97.84%)	1,081 (98.72%)	390 (97.50%)	
Yes	41 (2.16%)	14 (1.28%)	10 (2.50%)	
Arthritis, *n* (%)				<0.001
No	955 (50.29%)	683 (62.37%)	194 (48.50%)	
Yes	944 (49.71%)	412 (37.63%)	206 (51.50%)	
Chronic lung disease, *n* (%)				<0.001
No	1,622 (85.10%)	981 (89.59%)	322 (80.50%)	
Yes	284 (14.90%)	114 (10.41%)	78 (19.50%)	
Falls, *n* (%)				<0.001
No	1,231 (60.91%)	908 (82.92%)	308 (77.00%)	
Yes	790 (39.09%)	187 (17.08%)	92 (23.00%)	
Vision, *n* (%)				<0.001
Poor	476 (23.62%)	305 (27.85%)	110 (27.50%)	
Average	1,002 (49.73%)	491 (44.84%)	220 (55.00%)	
Good	537 (26.65%)	299 (27.31%)	70 (17.50%)	
Hearing, *n* (%)				<0.001
Poor	333 (16.52%)	218 (19.91%)	88 (22.00%)	
Average	1,147 (56.89%)	476 (43.47%)	227 (56.75%)	
Good	536 (26.59%)	401 (36.62%)	85 (21.25%)	
Self−perceived health status, *n* (%)				<0.001
Poor	589 (29.16%)	388 (35.43%)	107 (26.75%)	
Average	1,062 (52.57%)	530 (48.40%)	235 (58.75%)	
Good	369 (18.27%)	177 (16.16%)	58 (14.50%)	
Hip fracture, *n* (%)				<0.001
No	1909 (94.50%)	1,071 (97.81%)	372 (93.00%)	
Yes	111 (5.50%)	24 (2.19%)	28 (7.00%)	

ADL, activities of daily living; IADL, instrumental activities of daily living; SD, standard deviation; *n*, number. Percentages were calculated using non-missing values for each variable; therefore, category counts may not sum to the total cohort size because of missing data.

### 3.2 Model development and performance evaluation

Based on the 16 predictive variables identified through feature selection, this study constructed seven machine learning models to identify depressive symptoms in middle-aged and older adults with low haemoglobin. The overall performance of each model in the Development Cohort, Temporal Validation Cohort, and External Validation Cohort is shown in Figure 3 and Table 2. While Logistic Regression and GLMNet performed comparably in the Development Cohort, Random Forest demonstrated superior transportability and generalisation when challenged by the independent hospital-based external validation cohort. Furthermore, Random Forest inherently captures complex non-linear relationships and high-order feature interactions without pre-specifying functional forms. Therefore, Random Forest was retained for subsequent SHAP interpretation and online tool development.

**Figure 3:**
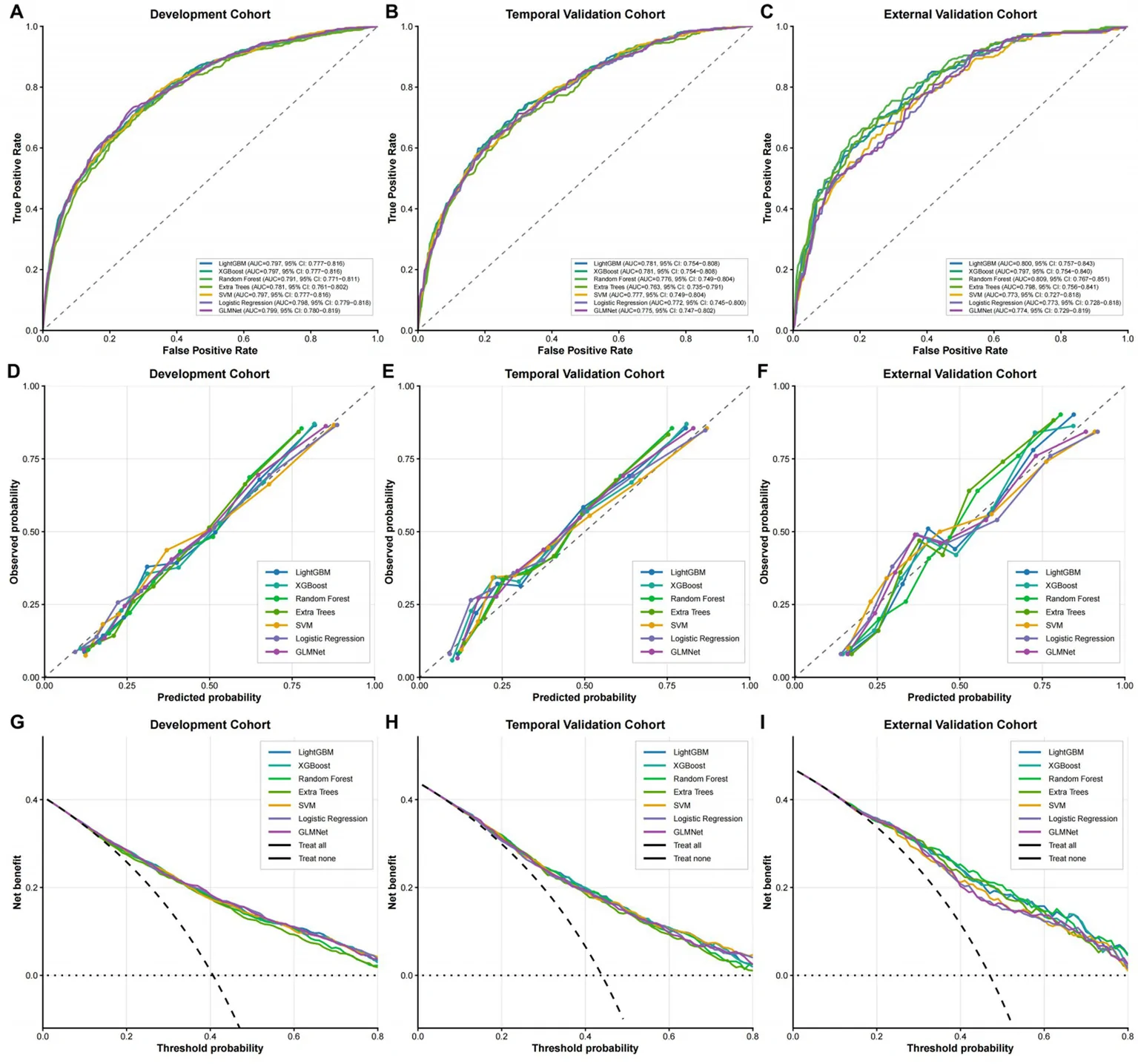
Model performance across three datasets. **(A–C)** ROC curves. **(D–F)** Calibration curves. **(G–I)** Decision curve analysis.

**Table 2:** Performance metrics of different machine learning models for identifying depressive symptoms in middle-aged and older adults with low haemoglobin across the development, temporal validation, and external validation cohorts.

Dataset	Metric	LightGBM	XGBoost	Random forest	Extra trees	SVM	Logistic regression	GLMNet
Development cohort	Accuracy	73.10%	72.80%	72.75%	72.01%	73.15%	74.09%	73.69%
	Precision	70.87%	69.60%	70.62%	70.19%	71.43%	72.99%	72.94%
	Specificity	83.83%	82.42%	83.92%	84.25%	84.50%	85.42%	85.75%
	F1 score	63.44%	63.72%	62.75%	61.13%	63.14%	64.35%	63.41%
	Recall	57.42%	58.76%	56.45%	54.14%	56.57%	57.54%	56.08%
	AUROC	0.797	0.797	0.791	0.781	0.797	0.798	0.799
	Brier score	0.178	0.178	0.182	0.186	0.178	0.177	0.177
Temporal validation cohort	Accuracy	71.32%	71.32%	70.87%	70.32%	70.87%	70.50%	70.32%
	Precision	74.63%	73.52%	73.96%	73.49%	72.63%	73.37%	74.07%
	Specificity	85.99%	84.69%	85.67%	85.67%	84.04%	85.34%	86.32%
	F1 score	61.71%	62.44%	61.05%	60.02%	61.98%	60.56%	59.63%
	Recall	52.60%	54.26%	51.98%	50.73%	54.05%	51.56%	49.90%
	AUROC	0.781	0.781	0.776	0.763	0.777	0.772	0.775
	Brier score	0.191	0.191	0.193	0.198	0.192	0.194	0.193
External validation cohort	Accuracy	71.50%	71.50%	73.50%	71.50%	69.25%	68.00%	69.25%
	Precision	72.29%	71.76%	76.62%	76.43%	71.24%	68.99%	71.52%
	Specificity	78.30%	77.36%	83.02%	84.43%	79.25%	76.89%	79.72%
	F1 score	67.80%	68.16%	69.01%	65.24%	63.93%	63.01%	63.72%
	Recall	63.83%	64.89%	62.77%	56.91%	57.98%	57.98%	57.45%
	AUROC	0.800	0.797	0.809	0.798	0.773	0.773	0.774
	Brier score	0.183	0.184	0.182	0.188	0.196	0.196	0.195

AUROC, area under the receiver operating characteristic curve; SVM, support vector machine; XGBoost, extreme gradient boosting; LightGBM, light gradient boosting machine; GLMNet, generalized linear model with elastic net regularization.

Specifically, the AUROC values for Random Forest in the Development Cohort, Temporal Validation Cohort, and External Validation Cohort were 0.791, 0.776, and 0.809, respectively, with corresponding accuracy values of 72.75%, 70.87%, and 73.50%, and Brier scores of 0.182, 0.193, and 0.182. Precision–recall analysis showed AUPRC values of 0.730, 0.734, and 0.794 in the Development Cohort, Temporal Validation Cohort, and External Validation Cohort, respectively. Calibration analysis showed generally acceptable calibration, with calibration intercepts of 0.010, 0.219, and 0.048, and calibration slopes of 1.262, 1.183, and 1.502, respectively, although some variation in calibration slope was observed across cohorts. Decision curve analysis suggested potential net benefit across a range of threshold probabilities, indicating possible clinical utility. In the hospital-based External Validation Cohort, model performance was not interpreted on the basis of AUROC alone; the Brier score, calibration intercept, calibration slope, and decision curve analysis were also considered to provide a more comprehensive assessment of external performance. Therefore, the external validation results suggest potential transportability of the model, but they should be interpreted together with calibration and clinical utility results rather than based solely on AUROC. Relevant calibration metrics are presented in Table 3, while the precision -recall curves and AUPRC results are shown in Supplementary Figure S2 and Supplementary Table S5. In the External Validation Cohort, predicted probabilities were generally higher among participants with depressive symptoms, and the observed prevalence of depressive symptoms increased progressively across risk groups, from 10.0% in the low-risk group (*n* = 40) to 32.0% in the medium-risk group (*n* = 206) and 76.6% in the high-risk group (*n* = 154). These findings support the exploratory risk-stratification ability of the model, but the thresholds should not be interpreted as clinically validated diagnostic cut-offs. The relevant results are presented in Supplementary Figures S3–S5. Threshold sensitivity analysis showed that lowering the classification threshold increased sensitivity/recall but reduced specificity. In the External Validation Cohort, sensitivity/recall increased from 62.8% at the default threshold of 0.50% to 92.0% at a threshold of 0.30, while specificity decreased from 83.0% to 40.6%. The threshold-dependent performance metrics are presented in Supplementary Table S5.

**Table 3:** Calibration intercept and calibration slope of the final random forest model across the development, temporal validation, and external validation cohorts.

Dataset	Calibration intercept	Calibration slope
Development cohort	0.010	1.262
Temporal validation cohort	0.219	1.183
External validation cohort	0.048	1.502

### 3.3 SHAP analysis of model interpretability

Figure 4 presents the results of the SHAP interpretability analysis for the final Random Forest model. Figures 4A,B show that Sleep time (hours), IADL score, ADL score, Life satisfaction, Arthritis, Cognitive score, Age, Self-perceived health status, Falls, and Gender were the top 10 key variables contributing to the model prediction, with Sleep time (hours), IADL score, and ADL score making the greatest contributions. Overall, these variables represented the main contributors to the model output in the external validation cohort. SHAP local explanations further illustrated how different predictors contributed to individual-level model predictions. Figure 4C presents the waterfall plot for a high-risk participant, in which the predicted probability increased from the baseline value of 0.406 to 0.904. The factors contributing most to the increased predicted probability were, in descending order, an IADL score of 3 (+0.122), Life satisfaction of 2 (+0.121), the combined contribution of other features (+0.0852), an ADL score of 2 (+0.0813), Self-perceived health status of 1 (+0.0578), and a Cognitive score of 2.5 (+0.0307). Figure 4D presents the waterfall plot for a low-risk participant, in which the predicted probability decreased from the baseline value of 0.406 to 0.114. The main factors contributing to the reduced predicted probability were the combined contribution of other features (−0.0792), a Cognitive score of 15.5 (−0.0516), an IADL score of 0 (−0.0459), Sleep time (hours) of 7 (−0.0413), absence of Arthritis (−0.0371), and an ADL score of 0 (−0.0367). Figure 5 further presents the SHAP dependence plots for the top nine key variables. The results showed that Sleep time (hours), Cognitive score, and Age showed non-linear contribution patterns in the model output; IADL score, ADL score, Life satisfaction, and Self-perceived health status showed relatively distinct hierarchical patterns, whereas Arthritis and Falls displayed discrete distribution patterns. Overall, the relationships between predictor variables and the model output were not simply linear, but instead reflected the coexistence of non-linear variation, hierarchical distribution, and discrete characteristics. These findings illustrate that the Random Forest model captured complex predictor patterns, thereby improving the transparency of model prediction. These SHAP findings describe predictor contributions to model outputs and should not be interpreted as evidence of causal pathways.

**Figure 4:**
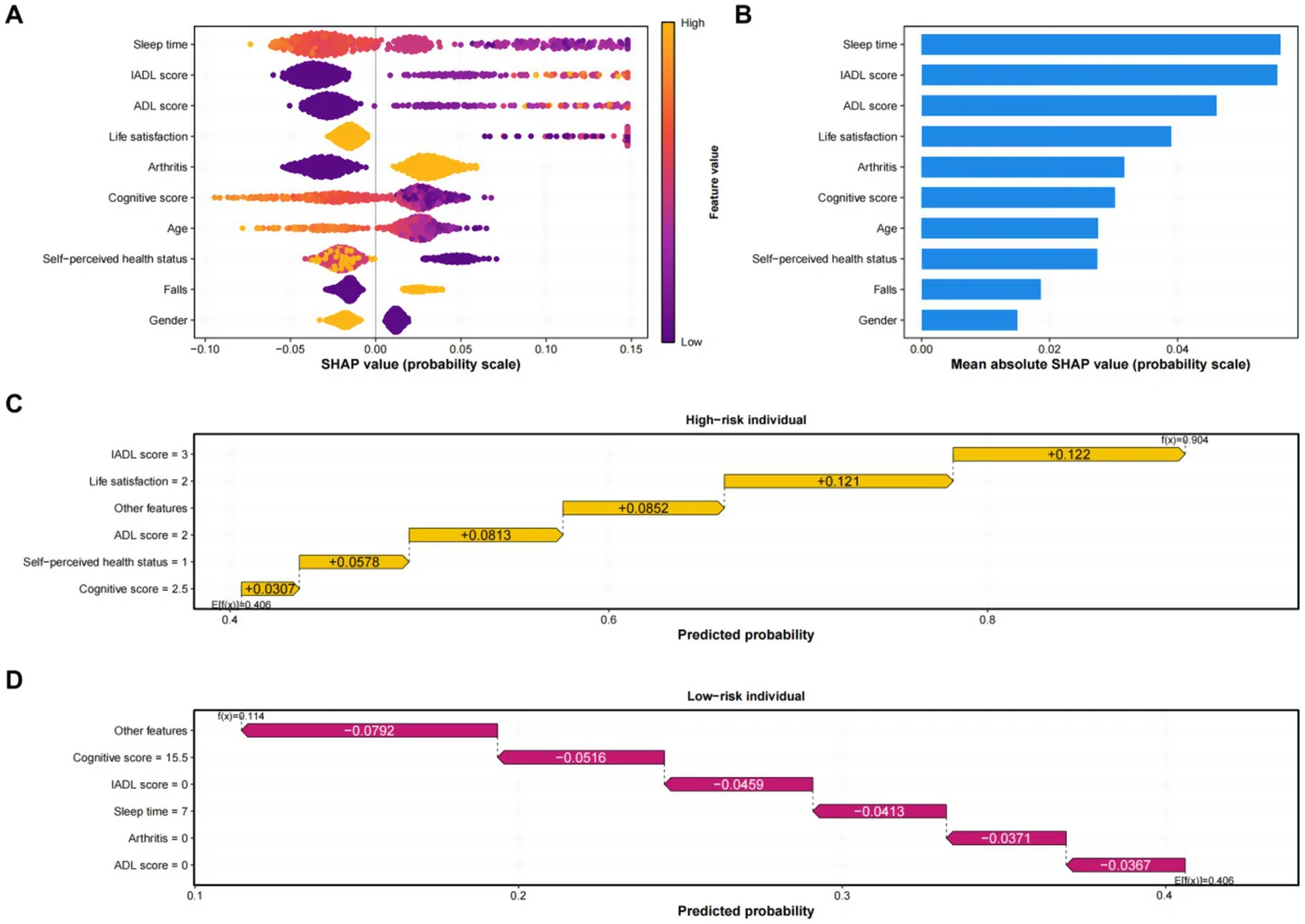
SHAP interpretation of the final Random Forest model for identifying depressive symptoms. **(A)** SHAP summary plot of the top 10 predictors in the external validation set. Each dot represents one participant; the *x*-axis shows the SHAP value, and the color represents the feature value. **(B)** Ranking of the top 10 predictors by mean absolute SHAP values. **(C)** Waterfall plot for a high-risk individual, showing contributions of each predictor. **(D)** Waterfall plot for a low-risk individual, showing contributions of each predictor.

**Figure 5:**
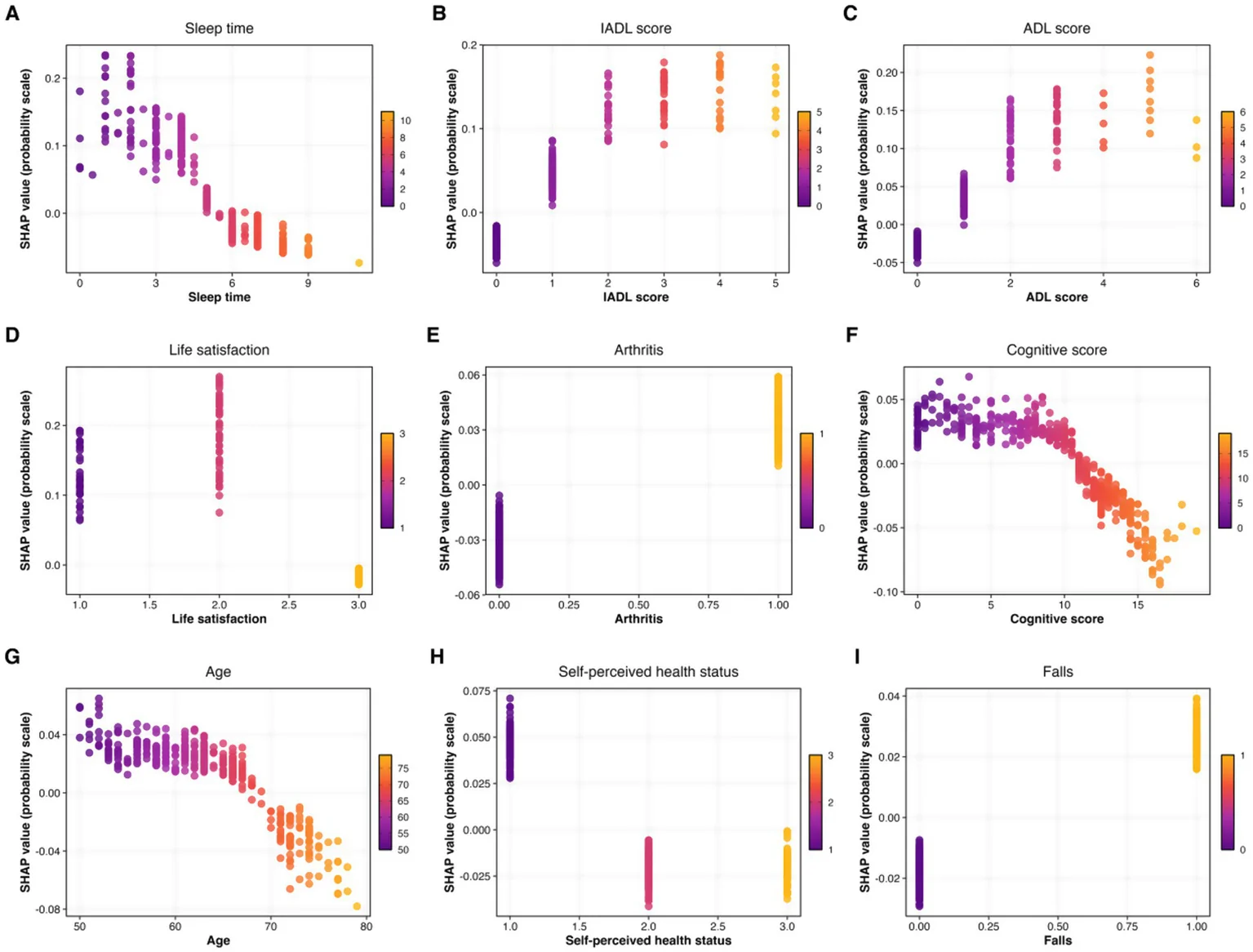
SHAP dependence plots for the top 9 predictors in the external validation set. Each panel shows the relationship between SHAP value (y-axis) and feature value (x-axis). Purple indicates lower feature values, whereas orange and yellow indicate higher feature values.

### 3.4 Development of a prototype online assessment tool

Based on the final Random Forest model, a prototype online assessment tool was developed and made publicly available at: https://anemia-older-depression.shinyapps.io/Anemia_Depression_Calculator/. The tool incorporates all 16 predictors in the final model and automatically outputs the predicted probability of depressive symptoms together with the corresponding exploratory risk stratification result after the relevant information is entered. The interface is shown in Figure 6.

**Figure 6:**
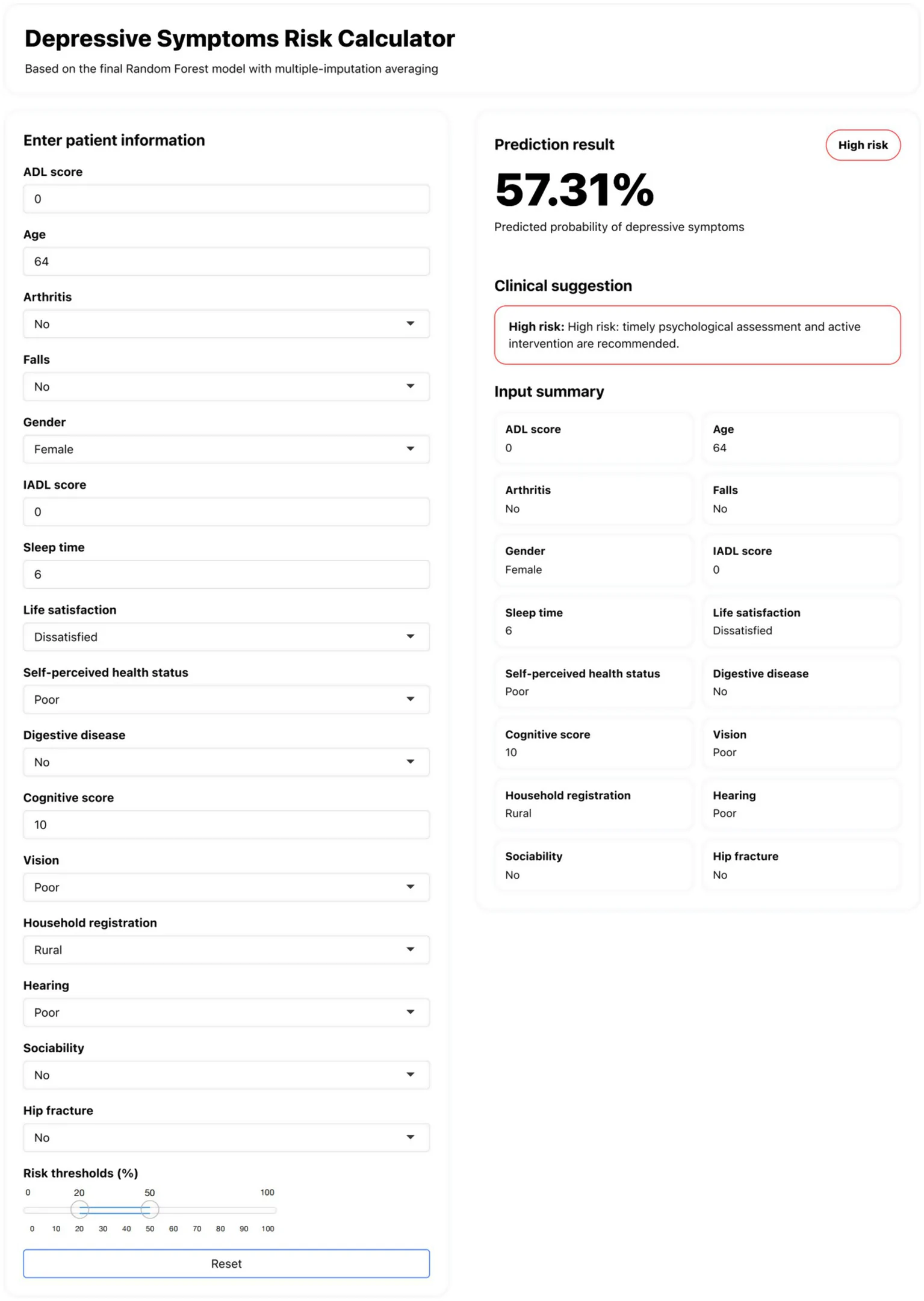
SHAP dependence plots for the top nine predictors in the External Validation Cohort. **(A)** Sleep time (hours); **(B)** IADL score; **(C)** ADL score; **(D)** Life satisfaction; **(E)** Arthritis; **(F)** Cognitive score; **(G)** Age; **(H)** Self-perceived health status; and **(I)** Falls. Each panel shows the relationship between SHAP value (probability scale; y-axis) and feature value (x-axis). Points are colored from purple (lower feature values) through pink/red to orange/yellow (higher feature values).

## 4 Discussion

To our knowledge, this study is the first to utilize the CHARLS database and an external clinical cohort to develop and validate machine learning models for identifying depressive symptoms in middle-aged and older adults with low haemoglobin. The results showed that the final model demonstrated relatively stable discrimination, generally acceptable calibration, and potential clinical utility across the three cohorts. The importance of this study lies in its focus on low haemoglobin as a routinely measurable, nutrition-related, and clinically relevant high-risk phenotype, providing an interpretable risk stratification tool to support early screening and stratified management in this vulnerable population.

It should be noted that the hospital-based external validation cohort differed from the CHARLS cohorts in education level, Internet use, sleep time, functional status, and comorbidity burden. These differences may affect model transportability and clinical interpretation. Therefore, the external validation results should not be interpreted solely based on AUROC, but should be considered together with calibration performance, Brier score, and decision curve analysis. These findings suggest preliminary external applicability, although further validation in larger, multicentre, and more diverse clinical populations is still needed.

From the perspectives of nutrition and clinical geriatrics, low haemoglobin may help define a routinely measurable, nutrition-related, and clinically relevant subgroup of middle-aged and older adults, as it may be associated with nutritional deficiency, inflammation-related processes, chronic disease burden, and frailty-related vulnerability (6, 29). In this specific subgroup, depressive symptoms are unlikely to occur in isolation, but may overlap with broader physical, functional, cognitive, and health-related vulnerabilities (30, 31). Therefore, developing a model to identify depressive symptoms in middle-aged and older adults with low haemoglobin may help identify individuals with an elevated probability of depressive symptoms and may have relevance for risk identification in a clinically and nutritionally vulnerable subgroup. The findings of this study are generally consistent with existing evidence and further refine the perspective on risk identification. Previous research has demonstrated a significant association between anaemia and depressive symptoms. Studies based on CHARLS have shown that depressive symptoms and anaemia are associated in both cross-sectional and longitudinal analyses, and that changes in depressive symptoms are associated with subsequent risk of anaemia, suggesting a possible dynamic bidirectional relationship between the two (32). The Canadian Study of Older Adults similarly reported a significant association between anaemia and depression, with an even higher risk of depression among those with untreated anaemia (33). Another longitudinal study found that the independent association between anaemia and depression was no longer significant after further adjustment for physical activity, functional status, and comorbidity, suggesting that functional status and comorbidity may partly explain the observed association between low haemoglobin and depressive symptoms (34). The findings of the present study are consistent with this observation: variables reflecting functional status and physical vulnerability, such as IADL, ADL, self-perceived health status, Falls, and Arthritis, all emerged as key predictors. This suggests that, among middle-aged and older adults with low haemoglobin, the estimated probability of depressive symptoms does not depend solely on haemoglobin levels but is more likely to be associated with functional capacity, chronic disease burden, sleep-related factors, and poorer subjective health perception. Beyond anaemia-related evidence, emerging evidence also supports the link between metabolic vulnerability and depression. Higher cardiovascular-kidney-metabolic syndrome stages have been associated with increased depression risk, particularly among older adults and other vulnerable sociodemographic groups (13). Metabolic syndrome has also been linked to a substantial and increasing burden of depressive symptoms in middle-aged and older adults (14), while adverse metabolic biomarker profiles, particularly higher glucose and triglyceride levels and lower high-density lipoprotein cholesterol levels, have been associated with future risks of depression, anxiety, and stress-related disorders (15). Taken together with the present findings, this broader evidence supports the view that the low-haemoglobin subgroup may represent a clinically and nutritionally relevant vulnerable population in which depressive symptom identification should consider metabolic, functional, comorbidity-related, and subjective health-related information.

In terms of key predictors, Sleep time (hours), IADL score, ADL score, Life satisfaction, Arthritis, Cognitive score, Age, Self-perceived health status, Falls, and Gender ranked among the top variables in the SHAP analysis. These findings should be interpreted primarily as model-based explanations of how routinely available predictors contributed to the estimated probability of depressive symptoms, rather than as evidence of new causal mechanisms. The identified predictors are largely consistent with established depression-related factors in middle-aged and older adults, including sleep time, functional impairment, subjective health status, cognitive function, and physical vulnerability indicators such as arthritis and falls. Previous studies have reported associations between sleep time and depressive symptoms (35), between ADL/IADL impairment and depressive symptoms (36), and between poor self-perceived health and depressive symptoms (37). Life satisfaction was also retained as an important model predictor, which may capture aspects of subjective well-being in this population. Previous research has also linked anaemia with cognitive decline among middle-aged and older adults (38), and falls may reflect increased physical vulnerability in older populations (39). Gender, self-perceived health, sleep, age, and cognitive function have also been identified as important predictive features in previous machine learning studies of depression among older adults (40–42). In the present study, SHAP analysis helped show that these known clinical, functional, and subjective health-related characteristics contributed to the model’s prediction process among individuals with low haemoglobin. While these predictors are well-established in the general population, their relative importance and joint predictive value had not been previously characterized in this vulnerable subgroup. Therefore, the main value of the SHAP results lies in improving model transparency, supporting clinical interpretability, and helping users understand individual-level risk profiles. However, because this study was observational and model-based, the SHAP results should not be interpreted as proving causal pathways between these predictors and depressive symptoms.

Among the seven machine learning models, Random Forest was retained for subsequent SHAP interpretation and online tool development because it showed relatively balanced overall predictive performance when discrimination, calibration, clinical utility, cross-cohort consistency, and interpretability were considered together. GLMNet and Logistic Regression showed slightly better AUROC and Brier score in the Development Cohort, but these advantages were small and were not consistently maintained in the validation cohorts. Therefore, Random Forest should be viewed as a pragmatic choice based on balanced performance and SHAP-based explainability, rather than as a model statistically superior to all alternatives. Specifically, the AUROC values for Random Forest in the Development Cohort, Temporal Validation Cohort, and External Validation Cohort were 0.791, 0.776, and 0.809, respectively, while the corresponding AUPRC values were 0.730, 0.734, and 0.794, indicating acceptable precision–recall performance across cohorts. The calibration intercept and calibration slope suggested a generally acceptable calibration, although some variation in calibration slope was observed across cohorts. As a bagging-based ensemble learning method, Random Forest constructs multiple decision trees through bootstrap sampling and random feature selection. It can capture complex non-linear relationships between variables while reducing the variance and overfitting risk associated with individual trees, making it suitable for high-dimensional and heterogeneous clinical data. Compared with traditional models based on linear assumptions, Random Forest offers greater flexibility in handling complex interactions; compared with some boosting-based models, it may provide relatively stable and interpretable predictions in heterogeneous clinical data, although further independent validation is still needed. Previous studies have also reported good performance of Random Forest in a range of medical prediction tasks (43, 44). Compared with previous machine learning studies on depression identification, this study has two notable features. First, the study population was more targeted. While previous studies have largely focused on the general middle-aged and older population, this study focused on middle-aged and older adults with low haemoglobin, a routinely measurable, nutrition-related, and clinically relevant high-risk phenotype; therefore, it may better capture the risk characteristics of this population (45). Second, the validation strategy was more comprehensive. In addition to the CHARLS Development Cohort, this study included the CHARLS Temporal Validation Cohort and an external hospital-based validation cohort, thereby evaluating model performance and preliminary external applicability in both community-based and clinical samples. Furthermore, SHAP analysis improved model transparency, and a prototype online assessment tool was developed to demonstrate the potential translation of the model into an individualised risk-estimation aid. Overall, this study provides an interpretable framework for screening depressive symptoms in middle-aged and older adults with low haemoglobin.

Regarding clinical utility, our model predicts CESD-10-defined depressive symptoms rather than clinically diagnosed major depression. In busy non-psychiatric or primary care settings, formal psychiatric diagnostic interviews are often impractical due to limited clinical time and resources. Because physical fatigue in older adults with low haemoglobin frequently masks depressive distress, our model offers a pragmatic, first-stage triage tool to prioritize high-risk patients for formal psychological assessment. In clinical or community settings, the proposed model should be used as an auxiliary tool for initial screening and risk stratification, rather than as a diagnostic instrument. The model may help clinicians and community health workers estimate depressive symptom probability among individuals with low haemoglobin and prioritize those who may require further CESD-10 screening, closer follow-up, or professional mental health evaluation. The SHAP-based output may also provide an interpretable risk profile by showing which predictors contribute most to an individual’s estimated risk, thereby supporting more targeted assessment and early supportive intervention. Because depressive symptom screening usually places greater emphasis on sensitivity and avoiding missed cases, the default 0.5 threshold should not be regarded as the only clinically relevant threshold. The threshold sensitivity analysis showed that lower thresholds improved sensitivity/recall at the cost of reduced specificity. Therefore, in screening-oriented settings where early identification is prioritized, a lower probability threshold may be considered to reduce missed cases, although this would increase false-positive classifications. Threshold selection should depend on the intended use, available clinical resources, and the acceptable balance between sensitivity and specificity. Individuals classified as medium or high risk, or otherwise identified as having elevated predicted risk, should receive further CESD-10 screening and, when necessary, professional psychiatric evaluation, rather than being diagnosed directly based on the model output. Because missing or inaccurate inputs may affect risk estimation, the online tool should be used with accurate and complete predictor information whenever possible. When substantial information is unavailable, the output should be interpreted with caution, and conventional depression screening and clinical assessment should be prioritized.

The online calculator should therefore be viewed as a prototype translation tool rather than a tool ready for routine clinical implementation. Before real-world clinical or community use, it requires prospective validation, routine calibration monitoring, privacy and data-security assessment, and workflow evaluation to determine how clinicians or community health workers interpret and act on the predicted risk output. Its results should be integrated with CESD-10 screening, follow-up assessment, and referral pathways rather than used as an independent clinical decision.

This study has several limitations that should be acknowledged. First, the model was developed primarily using CHARLS data, and some variables were self-reported, which may introduce recall and reporting bias. Second, the external validation cohort was recruited from a single hospital with a relatively small sample size, which inevitably restricts the generalisability and transportability of the findings. Although this cohort provided preliminary real-world validation across distinct clinical settings, the current results should be interpreted with caution. Future large-scale, prospective, multicentre studies across diverse healthcare tiers and geographic regions are needed to confirm the model’s broader clinical applicability. Third, specific biological and nutritional markers—including serum ferritin, albumin, inflammatory cytokines, and formal frailty scales—were not available across all cohorts. Although functional indicators served as practical proxies for frailty, the lack of detailed laboratory markers limits our ability to elucidate underlying biological mechanisms and etiology. Future prospective studies incorporating comprehensive biomarker panels are needed to delineate these pathophysiological pathways. Fourth, depressive symptoms were defined using the CESD-10 rather than structured clinical diagnostic interviews; therefore, this model cannot establish a formal clinical diagnosis of major depressive disorder and must be regarded strictly as an opportunistic pre-screening and triage aid rather than a diagnostic instrument. In addition, the prototype online calculator requires prospective validation and workflow evaluation before routine clinical implementation. Fifth, the threshold strategy should be interpreted with caution. The 0.5 classification threshold was used for conventional model performance reporting, and the risk-stratification cut-offs of <0.20, 0.20–<0.50, and ≥0.50 were exploratory rather than clinically validated thresholds. Although threshold sensitivity analysis was performed, optimal thresholds for clinical screening should be determined in future prospective and multicentre validation studies according to screening goals, sensitivity-specificity trade-offs, clinical resources, and follow-up evaluation pathways. Finally, as an observational and model-based study, this research does not allow causal inference. Although SHAP analysis was used to explain predictor contributions to the model output and improve model transparency, it should not be interpreted as evidence of causal relationships or biological mechanisms linking these predictors to depressive symptoms. In addition, because our primary focus was multivariable risk prediction using routinely available indicators, this study provides limited novel knowledge regarding previously unknown etiological risk factors.

## 5 Conclusion

This study used the CHARLS database and an external hospital-based validation cohort to develop and validate an interpretable machine learning model for identifying depressive symptoms in middle-aged and older adults with low haemoglobin. Among the seven candidate algorithms, Random Forest showed relatively balanced predictive performance across the three cohorts. The model incorporated demographic, health status, functional, cognitive, and comorbidity-related information, and may provide a useful reference for early screening and stratified management in this clinically vulnerable population. However, the default classification threshold and risk-stratification cut-offs should be interpreted as exploratory rather than clinically validated thresholds. Further multicentre validation is warranted before broader implementation, and future studies should determine optimal screening thresholds, evaluate the prototype online calculator in real-world workflows, and incorporate direct nutritional, inflammatory, and frailty-related indicators to further evaluate depressive symptom risk identification and underlying vulnerability profiles in this population.

## Data Availability

The raw data supporting the conclusions of this article will be made available by the authors, without undue reservation.
