# Supplementary material for: Development and external validation of an explainable machine learning model for identifying depressive symptoms in middle-aged and older adults with low haemoglobin: a prediction-model study
Source: Front Nutr. 2026 Oct 9;13:1846276. doi: 10.3389/fnut.2026.1846276 (PMC13690525; doi:10.3389/fnut.2026.1846276)
Supplement: Supplementary file 1 [file Supplementary_file_1.docx]

**Supplementary Tables**

**Table S1.** Sample size and outcome event distribution across cohorts.

| **Cohort** | **Total n** | **Non-depressive, n** | **Depressive, n** | **Event rate** |
| --- | --- | --- | --- | --- |
| Development cohort / CHARLS 2015 | 2,022 | 1,200 | 822 | 40.65% |
| Temporal validation cohort / CHARLS 2011 | 1,095 | 614 | 481 | 43.93% |
| External validation cohort | 400 | 212 | 188 | 47.00% |

Depressive symptoms were defined as a 10-item Centre for Epidemiologic Studies Depression Scale (CESD-10) score of 10 or higher. Abbreviation: n, number.

**Table S2.** Definitions and coding of candidate predictors.

| **Variable** | **Definition** |
| --- | --- |
| Age | Continuous variable in the model analysis. |
| Sleep time | Continuous variable in the model analysis. |
| Number of family members | Continuous variable in the model analysis. |
| IADL score | Continuous variable ranging from 0 to 5, calculated as the sum of five instrumental activities of daily living items (doing household chores, cooking, shopping, making phone calls, and taking medications). Each item was coded as 0/1 according to whether difficulty was present, with higher scores indicating poorer functional status. |
| ADL score | Continuous variable ranging from 0 to 6, calculated as the sum of six activities of daily living items (dressing, bathing, eating, getting in/out of bed, toileting, and continence control). Each item was coded as 0/1 according to whether difficulty was present, with higher scores indicating poorer functional status. |
| Cognitive score | Continuous variable ranging from 0 to 21, calculated as the sum of the Telephone Interview for Cognitive Status-10 (TICS-10) score (0-10; orientation and attention), word recall score (0-10; average of immediate and delayed recall, assessing episodic memory), and figure drawing score (0-1; visuospatial ability). Higher scores indicate better cognitive function. |
| Gender | 0 = Female, 1 = Male |
| Education | 0 = Below high school, 1 = High school, 2 = College or above |
| Marital status | 0 = Others (separated, divorced, widowed, or never married), 1 = Married or partnered (first married, remarried, or civil partner) |
| Household registration | 0 = Rural, 1 = Urban |
| Currently working | 0 = No, 1 = Yes |
| Internet use | 0 = No, 1 = Yes |
| Living with children | 0 = No, 1 = Yes |
| Smoking status | 0 = No (no current smoking), 1 = Yes (current smoking) |
| Alcohol consumption | 0 = No (not drinking now), 1 = Yes (drinking now) |
| Life satisfaction | Self-reported life satisfaction: 1 = Not satisfied, 2 = Average, 3 = Satisfied |
| Stroke | 0 = No, 1 = Yes |
| Sociability | Do you participate in social activities at least monthly? 0 = No, 1 = Yes |
| Liver diseases | Self-reported diagnosed liver disease by doctors: 0 = No, 1 = Yes |
| Kidney disease | Self-reported diagnosed kidney disease by doctors: 0 = No, 1 = Yes |
| Digestive disease | Self-reported diagnosed digestive disease by doctors: 0 = No, 1 = Yes |
| Diabetes | Self-reported diagnosed diabetes by doctors: 0 = No, 1 = Yes |
| Hypertension | Self-reported diagnosed hypertension by doctors: 0 = No, 1 = Yes |
| Dyslipidemia | Self-reported diagnosed dyslipidemia by doctors: 0 = No, 1 = Yes |
| Heart disease | Self-reported diagnosed heart disease by doctors: 0 = No, 1 = Yes |
| Diabetes medication use | 0 = No, 1 = Yes |
| Cancer | Self-reported diagnosed cancer by doctors: 0 = No, 1 = Yes |
| Arthritis | Self-reported diagnosed arthritis by doctors: 0 = No, 1 = Yes |
| Chronic lung disease | Self-reported diagnosed chronic lung disease by doctors: 0 = No, 1 = Yes |
| Falls | 0 = No, 1 = Yes |
| Vision | 1 = Poor, 2 = Average, 3 = Good |
| Hearing | 1 = Poor, 2 = Average, 3 = Good |
| Self-perceived health status | 1 = Poor, 2 = Average, 3 = Good |
| Hip fracture | 0 = No, 1 = Yes |

Abbreviations: ADL, activities of daily living; IADL, instrumental activities of daily living; TICS-10, Telephone Interview for Cognitive Status-10.

**Table S3.** Variance inflation factors (VIFs) for predictors included in the final Random Forest model.

| **Feature** | **VIF** |
| --- | --- |
| ADL score | 1.353 |
| IADL score | 1.332 |
| Cognitive score | 1.153 |
| Age | 1.129 |
| Self-perceived health status | 1.101 |
| Gender | 1.081 |
| Arthritis | 1.077 |
| Hearing | 1.063 |
| Vision | 1.061 |
| Digestive disease | 1.056 |
| Falls | 1.056 |
| Sleep time | 1.044 |
| Household registration | 1.031 |
| Life satisfaction | 1.028 |
| Sociability | 1.026 |
| Hip fracture | 1.025 |

Adjusted VIF values were calculated for categorical variables with multiple degrees of freedom. All VIF values were below 2, indicating no significant multicollinearity among predictors. Abbreviation: VIF, variance inflation factor.

**Table S4.** Threshold-dependent performance of the Random Forest model across different probability thresholds.

| **Cohort** | **Threshold** | **Accuracy** | **Sensitivity** | **Specificity** | **PPV** | **NPV** | **F1** |
| --- | --- | --- | --- | --- | --- | --- | --- |
| Development Cohort | 0.20 | 55.7% | 94.5% | 29.2% | 47.8% | 88.6% | 63.5% |
| Development Cohort | 0.30 | 67.3% | 85.2% | 55.1% | 56.5% | 84.4% | 67.9% |
| Development Cohort | 0.40 | 71.9% | 71.3% | 72.2% | 63.8% | 78.6% | 67.3% |
| Development Cohort | 0.50 | 72.7% | 56.4% | 83.9% | 70.6% | 73.8% | 62.7% |
| Temporal Validation Cohort | 0.20 | 60.5% | 93.8% | 34.4% | 52.8% | 87.6% | 67.6% |
| Temporal Validation Cohort | 0.30 | 66.8% | 80.2% | 56.2% | 58.9% | 78.4% | 68.0% |
| Temporal Validation Cohort | 0.40 | 70.3% | 68.2% | 72.0% | 65.6% | 74.3% | 66.9% |
| Temporal Validation Cohort | 0.50 | 70.9% | 52.0% | 85.7% | 74.0% | 69.5% | 61.1% |
| External Validation Cohort | 0.20 | 55.0% | 97.9% | 17.0% | 51.1% | 90.0% | 67.2% |
| External Validation Cohort | 0.30 | 64.8% | 92.0% | 40.6% | 57.9% | 85.1% | 71.0% |
| External Validation Cohort | 0.40 | 71.8% | 81.4% | 63.2% | 66.2% | 79.3% | 73.0% |
| External Validation Cohort | 0.50 | 73.5% | 62.8% | 83.0% | 76.6% | 71.5% | 69.0% |

Values are shown as percentages with one decimal place unless otherwise indicated. Threshold indicates the predicted probability cut-off used to classify participants as having depressive symptoms. Abbreviations: PPV, positive predictive value; NPV, negative predictive value.

**Table S5.** Area under the precision-recall curve (AUPRC) for the final Random Forest model in the development, temporal validation, and external validation cohorts.

| **Cohort** | **AUPRC** |
| --- | --- |
| Development cohort | 0.730 |
| Temporal validation cohort | 0.734 |
| External validation cohort | 0.794 |

Abbreviation: AUPRC, area under the precision-recall curve.

**Figure S1. Pearson correlation matrix of the final predictors included in the Random Forest model in the development cohort. The heatmap displays pairwise Pearson correlation coefficients among the predictors. Correlation coefficients are shown within each cell, with color intensity indicating the strength and direction of the association.**

**Figure S2.** Precision–recall curves of the final Random Forest model in the development, temporal validation, and external validation cohorts. The dashed horizontal line in each panel indicates the baseline prevalence of depressive symptoms.

**Figure S3. Confusion matrices of the final Random Forest model in the development, temporal validation, and external validation cohorts. The matrices present the numbers of true positives, true negatives, false positives, and false negatives using the default classification threshold of 0.5.**

**Figure S4. Distribution of predicted probabilities from the final Random Forest model according to depressive symptom status in the development, temporal validation, and external validation cohorts. Violin plots with embedded boxplots illustrate the separation of predicted risk probabilities between participants with and without depressive symptoms across the three datasets.**

**Figure S5. Risk stratification analysis of the final Random Forest model in the external validation cohort. (A) Distribution of predicted probabilities across predefined low-, medium-, and high-risk groups. (B) Observed depressive symptoms rate within each risk group together with the corresponding sample size.**
